# Long non-coding RNA profiling of pediatric Medulloblastoma

**DOI:** 10.1186/s12920-020-00744-7

**Published:** 2020-06-26

**Authors:** Varun Kesherwani, Mamta Shukla, Don W. Coulter, J. Graham Sharp, Shantaram S. Joshi, Nagendra K. Chaturvedi

**Affiliations:** 1 Child Health Research Institute, University of Nebraska Medical Center, Omaha, NE 69198 USA; 2 Department of Genetics, Cell Biology and Anatomy, University of Nebraska Medical Center, Omaha, NE 69198 USA; 3 Department of Pediatrics, Hematology and Oncology Division, University of Nebraska Medical Center, Omaha, NE 986395 USA; 4 Nebraska Medical Center, Omaha, NE USA

**Keywords:** Long non-coding RNA, Pediatric Medulloblastoma, Cancer biomarkers, Gene expression and pathways, Therapeutic targets

## Abstract

**Background:**

Medulloblastoma (MB) is one of the most common malignant cancers in children. MB is primarily classified into four subgroups based on molecular and clinical characteristics as (1) WNT (2) Sonic-hedgehog (SHH) (3) Group 3 (4) Group 4. Molecular characteristics used for MB classification are based on genomic and mRNAs profiles. MB subgroups share genomic and mRNA profiles and require multiple molecular markers for differentiation from each other. Long non-coding RNAs (lncRNAs) are more than 200 nucleotide long RNAs and primarily involve in gene regulation at epigenetic and post-transcriptional levels. LncRNAs have been recognized as diagnostic and prognostic markers in several cancers. However, the lncRNA expression profile of MB is unknown.

**Methods:**

We used the publicly available gene expression datasets for the profiling of lncRNA expression across MB subgroups. Functional analysis of differentially expressed lncRNAs was accomplished by Ingenuity pathway analysis (IPA).

**Results:**

In the current study, we have identified and validated the lncRNA expression profile across pediatric MB subgroups and associated molecular pathways. We have also identified the prognostic significance of lncRNAs and unique lncRNAs associated with each MB subgroup.

**Conclusions:**

Identified lncRNAs can be used as single biomarkers for molecular identification of MB subgroups that warrant further investigation and functional validation.

## Background

Medulloblastoma (MB), the most common pediatric brain tumor, constitutes nearly 20% of newly diagnosed brain tumors in children [1, 2]. Treatment of MB involves radiation therapy, chemotherapy and surgical resection. These strategies have improved the survival by 70–80% but also lead to serious morbidities [3, 4]. MB are classified into four major molecular subgroups as WNT, Sonic hedgehog (SHH), Group 3 and Group 4. The WNT subgroup is least common among all 4 subgroups and present in only 10% of cases. Genetic changes in genes: *CTNNB1*, *DDX3X*, *SMARCA4* and *DKK1* are frequently observed in the WNT subgroup. WNT has the best prognosis among all types of MB. SHH is second most common subgroup with abnormalities in SHH signaling pathway and accounts for ~ 30% of total MB cases. Genetic anomalies in genes: *MYCN*, *GLI1*, *PTCH1*, *SUFU*, *MLL2*, *SMO*, *TP53*, *BCOR1*, *GAB1*, *GABRG1* and *LDB1* are frequently seen in the SHH subgroup. The SHH subgroup has an intermediate prognosis among MB subgroups. Group 3 is the third most common subgroup with 25% of the total MB cases. Group 3 is mainly MYC-driven and genetic aberrations are seen in genes: *MYC*, *PVT1*, *OTX2*, *MLL2*, *SMARCA4*, and *CHD7* in this subgroup. The prognosis of the Group 3 is very poor and 5 year overall survival is less than 50%. Group 4 is the most common subgroup of MB and accounts for 35% of total cases. The prognosis of the Group 4 is intermediate and genetic aberrations are commonly present in genes: *OTX2*, *DDX31*, *CHD7*, *NCAIP*, *MYCN*, *CDK6*, *GFI1*/*GFI1B*, *MLL2*, *KDM6A*, *MLL3*, and *ZMYM3* [5–9]. Molecular markers used for WNT identification are CTNNB1 (nuclear), FLIA, YAP1 and DKK1; for SHH are SFRP1, GLI1, FLIA, YAP1 and GAB1; for Group 3, NPR3; and for Group 4, KCNA1. Identification of new molecular markers for drug targeting, diagnosis and prognosis are important due to need for improved molecular profiling of MB [10].

Long non-coding RNAs (LncRNAs) are RNAs of more than 200 bp in length and can be transcribed from an intergenic region, genic regions or super enhancer regions in the genome. LncRNAs can modulate chromatin structure, gene regulation via interactions with epigenetic modifiers and transcriptional co-factors, and also have post-translation effects via affecting the stability of mRNA or proteins [11, 12]. Deregulated lncRNA expression is associated with many cancers [13]. LncRNA signatures have been used to classify different types of cancer as biomarkers for diagnosis, prognosis and therapy [14–18]. LncRNAs are secreted in serum, plasma, and CSF in a stable form protected from endogenous RNAase and can be used for non-invasive analysis from patient samples [19, 20]. The role of lncRNA in brain development is well studied [21–26]. However, there is not much known about role of lncRNAs in MB. LncRNA LOXL1-AS1 promotes the proliferation and metastasis of MB by activating the PI3K-AKT pathway [27]. LncRNA CCAT1 promotes cell proliferation and metastasis in human MB by regulating the MAPK pathway [28]. Silencing of ANRIL in MB cell lines significantly lowered cell viability and migration. ANRIL promoted the apoptosis of MB cell lines through miR-323-mediated regulation of BRI3, which activates p38 MAPK, ERK, and AKT as well as the WNT signaling pathway [29]. LINC-NeD125 expression is upregulated in Group 4 MB and after interacting to miRNA-induced silencing complex(MISC), it directly binds to miR-19a-3p, miR-19b-3p and miR-106a-5p. Functionally, LINC-NeD125 acts by sequestering the three miRNAs, which leads to the de-repression of major driver genes (*CDK6*, *MYCN*, *SNCAIP*, and *KDM6A*) of Group 4 MB [30]. LncRNA CRNDE expression is elevated in MB and knockdown of CRNDE significantly reduced cell proliferation and inhibited colony formation in MB cell lines, Daoy and D341 [31].

In the current study, we have identified the lncRNAs expression profile of pediatric MB subgroups and associated molecular pathways. We have also identified the unique lncRNAs associated with each subgroup.

## Methods

We searched the Gene Expression Omnibus (GEO) database for MB related microarray datasets and found two relevant studies, GSE37418 [for pediatric MB subgroups expression data] and GSM1094863, GSM1094864, GSM1094865, GSM1094866, GSM1094867 [for pediatric primary cerebellum expression data from GSE44971] for our analyses. We further used large GSE124814 datasets for the validation of lncRNAs expression profiles of MB subgroups obtained from our original analyses. We selected the age < 18 years as an inclusion criteria for selecting pediatric MB samples. We selected the datasets which used the Affymetrix U133 Plus2 array for probe level RNA expression studies. For data analyses, we first did background correction, normalization (RMA), quality control checks, intensity and batch effect corrections of each dataset. Following that, we did probe level differential analyses of datasets using the limma package (ANOVA with eBayes) with criteria of *p* < 0.001 and fold change greater than two folds. We then annotated the probe sets with the Affymetrix U133 Plus2 library and filtered out lncRNA genes. The lncRNA gene database used is verified and approved by HGNC. Functional analysis of differentially expressed lncRNAs was done by Ingenuity pathway analysis (IPA) software from BioRad, Inc. We used default parameters and checked all the node types, all species (except uncharted), and all tissue types for core analysis in IPA.

## Results

### Differentially expressed lncRNAs in the WNT subgroup and their functional roles

**Fig. 1:**
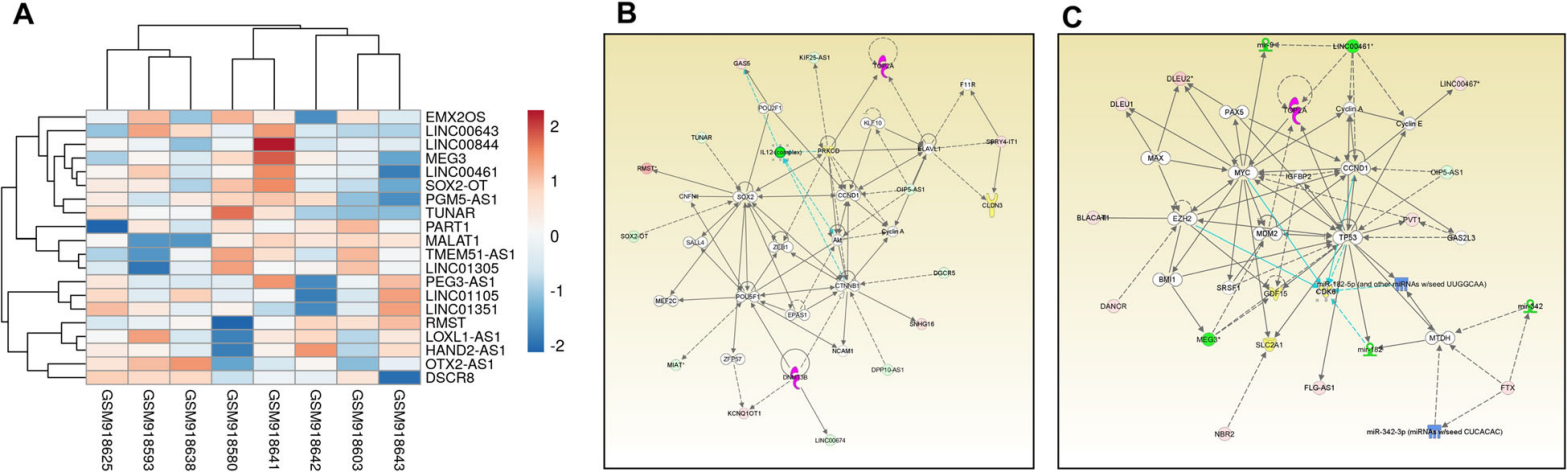
**a**: Heatmap of top 10 upregulated and downregulated lncRNAs in WNT subgroup. Expression value of different lncRNAs was clustered using correlation distance method. **b**: Differentially expressed lncRNAs in a non-canonical biological network in WNT subgroup. The important nodes in this biological network are CCND1, AKT, SOX2, POU5F1, DNMT3B, and CTNNB1. **c**: Differentially expressed lncRNAs in another non-canonical biological network in WNT subgroup. The important nodes in this biological network are TP53, MYC, EZH2, and MDM2. Green indicates downregulated and red indicates upregulated lncRNAs

Comparative analyses of WNT MB (*N* = 8) and normal cerebellum tissue (*N* = 5) datasets with *p* < 0.05 and fold changes > 2 provided 199 differentially expressed lncRNAs with approved status. Tables 1 and 2 show the fold change in the top 10 upregulated and downregulated lncRNAs. Heatmap of top 10 upregulated and downregulated lncRNAs is shown in Fig. 1a. The complete list of lncRNAs can be seen in Additional file 1. We found 73% overlap with lncRNAs in validation datasets [WNT *N* = 31, Control = 5] (Additional file 2). We found all the top 10 upregulated and downregulated lncRNAs present in validation datasets. We mostly see non-overlap in lncRNAs at lower expression values.

**Table 1:** Top 10 up-regulated lncRNAs in WNT subgroup of MB

Gene Symbol	Fold Change	P-val	FDR P-val
EMX2OS	38.18	9.01E-14	4.92E-10
OTX2-AS1	37.84	1.14E-09	3.93E-07
PGM5-AS1	30.26	9.54E-09	1.63E-06
DSCR8	24.56	0.0001	0.0013
LOXL1-AS1	21.06	1.03E-08	1.73E-06
HAND2-AS1	18.51	9.37E-07	3.59E-05
TMEM51-AS1	16.9	3.88E-09	8.82E-07
RMST	13.92	1.14E-07	8.49E-06
LINC01305	11.11	0.0001	0.001
PART1	10.94	1.89E-05	0.0003

**Table 2:** Top 10 down-regulated lncRNAs in WNT subgroup of MB

Gene Symbol	Fold Change	*P*-val	FDR *P*-val
LINC00461	−62.39	1.16E-06	4.17E-05
MEG3	−58.9	9.41E-07	3.60E-05
LINC00844	−24.94	0.0003	0.0024
LINC00643	−13.3	6.95E-06	0.0001
SOX2-OT	−12.13	0.0003	0.0024
PEG3-AS1	−10.02	3.49E-07	1.84E-05
TUNAR	−7.72	2.04E-12	5.87E-09
MALAT1	−7.39	1.65E-07	1.10E-05
LINC01105	−7.39	3.77E-05	0.0005
LINC01351	−6.48	2.24E-05	0.0003

We did functional analysis of differentially expressed (DE) lncRNAs of the WNT subgroup using IPA. We identified different functional parameters involved in this subgroup. MAX (a MYC interacting partner), miR-150, miR-133a, FOLR1, E2F NCAM1, GAS2L3 and ATF5 are the most significantly associated upstream regulators, while cancer, neurogenesis, metastasis and cellular development are the most important biological functions affected in this subgroup (Tables 3 and 4). Heatmap of 5 upstream regulators is shown in supplementary Fig. 1 (Additional file 3). The two most important non-canonical networks enriched with DE lncRNAs are shown in Fig. 1b and c. In networks 1; CCND1, AKT, SOX2, POU5F1, DNMT3B, and CTNNB1, in network 2; TP53, MYC, EZH2, and MDM2 are the central regulators linked with DE lncRNAs.

**Table 3:** Top 10 upstream regulators involved in DE lncRNAs in WNT subgroup

Upstream Regulator	Molecule Type	*P*-val of overlap	Target molecules in dataset
MAX	transcription regulator	5.53E-03	DLEU1,DLEU2
miR-150-5p (and other miRNAs w/seed CUCCCAA)	mature microRNA	5.54E-03	MIAT
miR-133a-3p (and other miRNAs w/seed UUGGUCC)	mature microRNA	7.38E-03	MALAT1
mir-133	microRNA	9.22E-03	MALAT1
FOLR1	transporter	1.31E-02	GAS5,PVT1
E2f	group	1.37E-02	DLEU1,DLEU2
ATF5	transcription regulator	1.47E-02	GAS5
NCAM1	other	1.84E-02	MALAT1
mir-150	microRNA	2.56E-02	MIAT
GAS2L3	other	2.92E-02	PVT1

**Table 4:** Top 10 disease and function identified by IPA from DE lncRNAs in WNT subgroup

Categories	Diseases or Functions Annotation	*P*-val	Activation z-score
Cellular Development, Cellular Growth and Proliferation, Nervous System Development and Function	Neurogenesis of nervous tissue cell lines	3.38E-06	
Cellular Movement	Cell movement of tumor cell lines	1.12E-05	1.324
Cellular Movement	Migration of tumor cell lines	1.14E-05	1.498
Cellular Movement	Invasion of tumor cell lines	1.55E-04	1.083
Cell Cycle	Arrest in G0 phase of tumor cell lines	3.83E-04	
Cancer, Organismal Injury and Abnormalities	Metastasis of tumor cell lines	4.26E-04	−0.277
Cell Death and Survival	Cell death of eye cell lines	5.07E-04	
Cellular Movement	Migration of cells	6.27E-04	0.573
Cellular Movement	Cell movement	6.75E-04	0.453
Cellular Movement	Migration of hepatoma cell lines	1.34E-03	

### Differentially expressed lncRNAs in the SHH subgroup and their functional roles

**Fig. 2:**
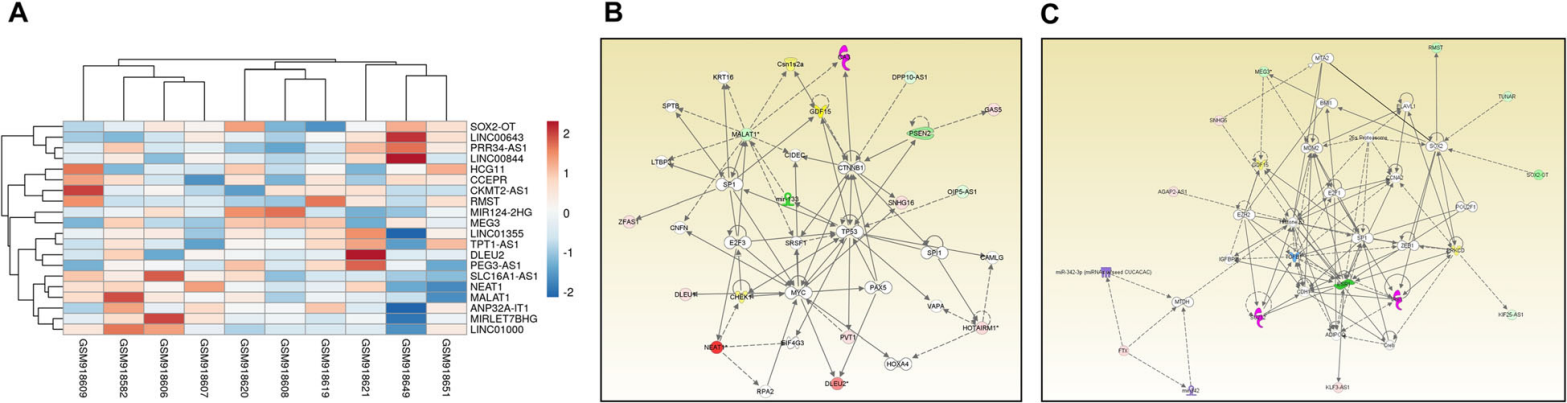
**a**: Heatmap of top 10 upregulated and downregulated lncRNAs in SHH subgroup. Expression value of different lncRNAs was clustered using correlation distance method. **b**: Differentially expressed lncRNAs in a non-canonical biological network in SHH subgroup. The important nodes in this biological network are CCND1, TP53, MYC, MALAT1, CTNNB1, and SP1. **c**: Differentially expressed lncRNAs in another non-canonical biological network in SHH subgroup. The important nodes in this biological network are Histone H3, MDM2, CCNA2, SOX2, POU2F1, SP1, and ESR1. Green indicates downregulated and red indicates upregulated lncRNAs

Comparative analyses of the SHH subgroup (*N* = 10) and normal cerebellum tissue (*N* = 5) datasets with *p* < 0.05 and fold change > 2 provided 145 differentially expressed lncRNAs with approved status. Tables 5 and 6 show the fold change in the top 10 upregulated and downregulated lncRNAs. Heatmap of top 10 upregulated and downregulated lncRNAs is shown in Fig. 2a. The complete list of lncRNAs can be seen in Additional file 1. We found 50% overlap with lncRNAs in validation datasets [SHH *N* = 65, Control = 5] (Additional file 2). We found all the top 10, upregulated and downregulated lncRNAs, present in validation datasets except DLEU2 and PRR34-AS1.

**Table 5:** Top 10 up-regulated lncRNAs in SHH subgroup of MB

Gene Symbol	Fold Change	*P*-val	FDR *P*-val
NEAT1	23.48	0.0003	0.0022
DLEU2	13.24	5.79E-11	2.41E-08
PRR34-AS1	8.07	1.58E-07	8.49E-06
LINC01355	8.05	2.93E-09	5.15E-07
MIRLET7BHG	7.49	1.79E-07	9.34E-06
CKMT2-AS1	6.23	1.86E-09	3.59E-07
SLC16A1-AS1	5.65	8.13E-08	5.36E-06
TPT1-AS1	5.28	4.44E-08	3.50E-06
LINC01000	4.96	1.10E-08	1.32E-06
ANP32A-IT1	4.94	9.36E-07	3.07E-05

**Table 6:** Top 10 down-regulated lncRNAs in SHH subgroup of MB

Gene Symbol	Fold Change	*P*-val	FDR *P*-val
LINC00844	−33.36	1.08E-08	1.32E-06
MIR124-2HG	−28.13	0.0005	0.0032
SOX2-OT	−13.83	2.39E-07	1.15E-05
PEG3-AS1	−12.94	5.12E-08	3.88E-06
LINC00643	−11.76	3.97E-06	8.83E-05
HCG11	−11.26	0.0012	0.0065
RMST	−9.43	0.0036	0.0155
CCEPR	−8.93	1.49E-06	4.27E-05
MEG3	−8.53	0.0002	0.0018
MALAT1	−8.25	2.82E-06	6.86E-05

Functional analysis of DE lncRNAs of SHH MB subgroup using IPA predicts, MAX (a MYC interacting partner), miR-133a, FOLR1, E2F, ATF5, AM1, E2F3, GAS2L3 and ACSL5 as most significantly associated upstream regulators, while cancer, neurogenesis, cell proliferation, metastasis and cellular development are the most important biological functions affected in this subgroup (Tables 7 and 8). Heatmap of 5 upstream regulators is shown in supplementary Fig. 1 (Additional file 3). The two most important non-canonical networks enriched with DE lncRNAs are shown in Fig. 2b and c. In network 1; CCND1, TP53, MYC, MALAT1, CTNNB1, and SP1, in network 2; Histone H3, MDM2, CCNA2, SOX2, POU2F1, SP1, and ESR1 are the central regulators linked with DE lncRNAs.

**Table 7:** Top upstream regulators involved in DE lncRNAs in SHH subgroup

Upstream Regulator	Molecule Type	*P*-val of overlap	Target molecules in dataset
MAX	transcription regulator	2.74E-03	DLEU1,DLEU2
miR-133a-3p (and other miRNAs w/seed UUGGUCC)	mature microRNA	5.17E-03	MALAT1
mir-133	microRNA	6.46E-03	MALAT1
FOLR1	transporter	6.58E-03	GAS5,PVT1
E2f	group	6.85E-03	DLEU1,DLEU2
ATF5	transcription regulator	1.03E-02	GAS5
NCAM1	other	1.29E-02	MALAT1
E2F3	transcription regulator	1.49E-02	MALAT1,NEAT1
GAS2L3	other	2.05E-02	PVT1
ACSL5	enzyme	2.18E-02	ST7-AS1

**Table 8:** Top 10 disease and function identified by IPA from DE lncRNAs in SHH subgroup

Categories	Diseases or Functions Annotation	*P*-val	Activation z-score
Cellular Development, Cellular Growth and Proliferation, Nervous System Development and Function	Neurogenesis of nervous tissue cell lines	1.79E-06	
Cellular Development, Cellular Growth and Proliferation	Proliferation of kidney cancer cell lines	3.01E-06	−0.095
Cellular Development, Cellular Growth and Proliferation	Cell proliferation of tumor cell lines	3.90E-04	0.933
Cellular Movement	Migration of carcinoma cell lines	5.25E-04	0.762
Cellular Movement	Migration of kidney cancer cell lines	6.63E-04	
Cellular Movement	Cell movement of tumor cell lines	6.65E-04	0.751
Cellular Movement	Migration of tumor cell lines	1.14E-03	1.033
Cellular Development, Cellular Growth and Proliferation	Cell proliferation of carcinoma cell lines	1.30E-03	0.277
Cellular Development, Connective Tissue Development and Function, Tissue Development	Osteogenic differentiation of nucleus pulposus cells	1.36E-03	
Cancer, Gastrointestinal Disease, Organismal Injury and Abnormalities	Stage I colorectal adenocarcinoma	1.36E-03	

### Differentially expressed lncRNAs in the Group 3 subgroup and their functional roles

**Fig. 3:**
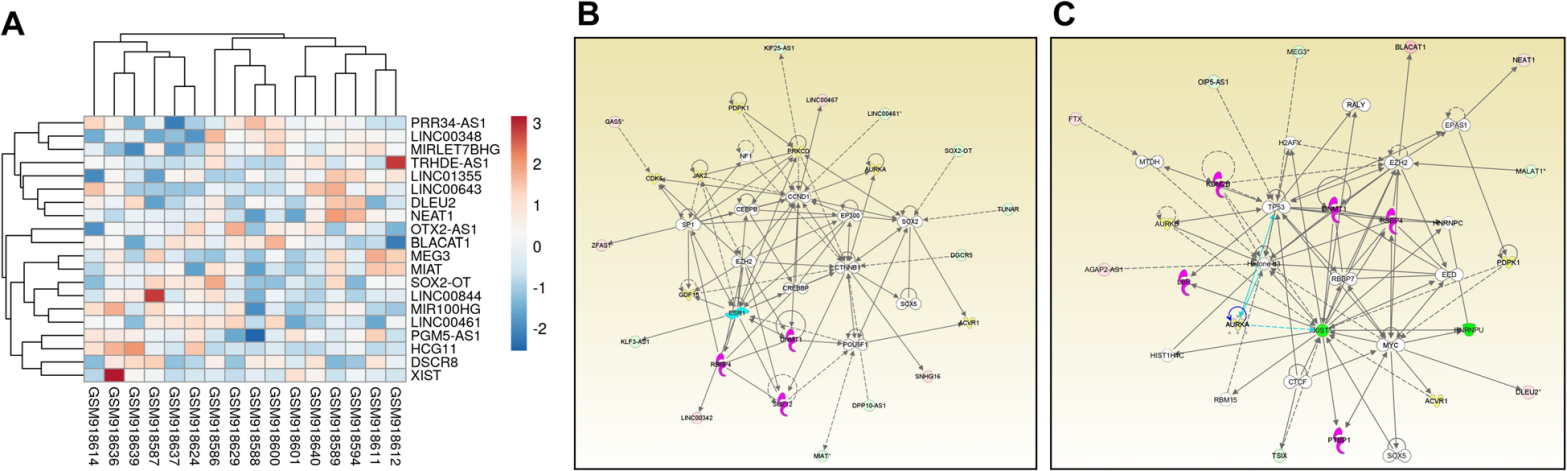
**a**: Heatmap of top 10 upregulated and downregulated lncRNAs in Group 3 MB. Expression value of different lncRNAs was clustered using correlation distance method. **b**: Differentially expressed lncRNAs in a non-canonical biological network in Group 3 MB. The important nodes in this biological network are CCND1, EP300, CREBBP, ESR1, CTNNB1, and PRKCD. **c**: Differentially expressed lncRNAs in another non-canonical biological network in Group 3 MB. The important nodes in this biological network are Histone H3, TP53, MYC, XIST, and EZH2. Green indicates downregulated and red indicates upregulated lncRNAs

Comparative analyses of the Group 3 MB (*N* = 16) and normal cerebellum tissue (*N* = 5) datasets with *p* < 0.05 and fold change > 2 provided 149 differentially expressed lncRNAs with approved status. Tables 9 and 10 show the fold change in the top 10 upregulated and downregulated lncRNAs. Heatmap of top 10 upregulated and downregulated lncRNAs is shown in Fig. 3a. The complete list of lncRNAs can be seen in Additional file 1. We found 86% overlap with lncRNAs in validation datasets [Group 3 *N* = 46, Control N = 5] (Additional file 2). We found all the top 10 upregulated and downregulated lncRNAs in the validation dataset, except NEAT1.

**Table 9:** Top 10 up-regulated lncRNAs in Group 3 of MB

Gene Symbol	Fold Change	*P*-val	FDR *P*-val
OTX2-AS1	80.96	1.03E-14	1.38E-11
BLACAT1	24.59	6.32E-08	3.32E-06
LINC00348	19.06	0.0013	0.0077
LINC01355	9.91	1.54E-08	1.09E-06
DLEU2	8.97	2.31E-09	2.49E-07
PGM5-AS1	7.73	4.32E-05	0.0005
NEAT1	7.27	0.0082	0.0315
DSCR8	6.73	0.0104	0.0378
PRR34-AS1	6.63	1.37E-06	3.58E-05
MIRLET7BHG	6.32	0.0004	0.003

**Table 10:** Top 10 down-regulated lncRNAs in Group 3 of MB

Gene Symbol	Fold Change	*P*-val	FDR *P*-val
XIST	−315.1	0.0066	0.0267
MEG3	−58.01	0.0001	0.0011
SOX2-OT	−50.3	1.15E-14	1.44E-11
LINC00844	−37.6	6.71E-08	3.47E-06
MIR100HG	−16.61	1.16E-06	3.15E-05
HCG11	−12.99	1.93E-05	0.0003
MIAT	−10.36	0.0002	0.0016
LINC00461	−9.38	0.002	0.0105
LINC00643	−9.34	0.0005	0.0036
TRHDE-AS1	−7.59	7.27E-09	6.14E-07

Functional analysis of DE lncRNAs of Group 3 MB using IPA predicted C17orf98, ZNF426, RNF165, FBXO8, CTCF, LAYN, PYGO1, Firre, TSIX and miR-150-5pa as most significantly associated upstream regulators, while activation/inactivation of X-chromosome, cell movement, and metastasis are the most important biological functions affected in this subgroup (Tables 11 and 12). Heatmap of 5 upstream regulators is shown in supplementary Fig. 2 (Additional file 3). The two most important non-canonical networks enriched with DE lncRNAs are shown in Fig. 3b and c. In network 1; CCND1, EP300, CREBBP, ESR1, CTNNB1, and PRKCD, in network 2; Histone H3, TP53, MYC, XIST, and EZH2 are the central regulators linked with DE lncRNAs.

**Table 11:** Top 10 upstream regulators involved in DE lncRNAs in Group 3 MB

Upstream Regulator	Molecule Type	*P*-val of overlap	Target molecules in dataset
C17orf98	other	1.11E-03	XIST
ZNF426	transcription regulator	1.11E-03	XIST
RNF165	enzyme	1.11E-03	XIST
FBXO8	other	1.11E-03	XIST
CTCF	transcription regulator	2.08E-03	TSIX,XIST
LAYN	other	2.22E-03	XIST
PYGO1	other	2.22E-03	XIST
Firre	other	3.33E-03	XIST
TSIX	other	3.33E-03	XIST
miR-150-5p (and other miRNAs w/seed CUCCCAA)	mature microRNA	3.33E-03	MIAT

**Table 12:** Top 10 disease and function identified by IPA from DE lncRNAs in Group 3 MB

Categories	Diseases or Functions Annotation	*P*-val	Activation z-score
Gene Expression	Inactivation of mouse X chromosome	5.74E-06	
Gene Expression	Activation of mouse X chromosome	5.74E-06	
Cellular Movement	Cell movement of tumor cell lines	2.36E-05	0.783
Cellular Movement	Migration of tumor cell lines	3.60E-05	0.955
Cell Cycle	Arrest in G0 phase of tumor cell lines	1.65E-04	
Cellular Movement	Invasion of tumor cell lines	6.99E-04	0.495
Cellular Movement	Cell movement	1.30E-03	0.804
Gene Expression	Imprinting	1.31E-03	
Cancer, Organismal Injury and Abnormalities	Metastasis of tumor cell lines	1.32E-03	0.152
Hereditary Disorder, Organismal Injury and Abnormalities	Familial skewed X inactivation	1.41E-03	

### Differentially expressed lncRNAs in the Group 4 MB and their functional roles

**Fig. 4:**
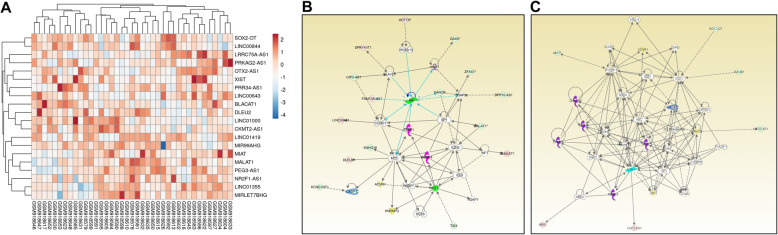
**a**: Heatmap of top 10 upregulated and downregulated lncRNAs in Group 4 MB. Expression value of different lncRNAs was clustered using correlation distance method. **b**: Differentially expressed lncRNAs in a non-canonical biological network in Group 4 MB. The important nodes in this biological network are AR, MYC, XIST, SP1, CCND1, and EZH2. **c**: Differentially expressed lncRNAs in another non-canonical biological network in Group 4 MB. The important nodes in this biological network are Histone H3, SP1, ESR1, MYC, SOX2, POU5F1, CDH1, and CEBPB. Green indicates downregulated and red indicates upregulated lncRNAs

Comparative analyses of Group 4 MB (*N* = 39) and normal cerebellar tissue (*N* = 5) datasets with *p* < 0.05 and fold change > 2 provided 150 differentially expressed lncRNAs with approved status. Tables 13 and 14 show the fold change in the top 10 upregulated and downregulated lncRNAs. Heatmap of top 10 upregulated and downregulated lncRNAs is shown in Fig. 4a. The complete list of lncRNAs can be seen in Supplementary file 1. We found 82% overlap with lncRNAs in validation datasets [Group 4 *N* = 95, Control = 5] (Additional file 2). We found all the top 10 upregulated and downregulated lncRNAs in validation datasets.

**Table 13:** Top 10 up-regulated lncRNAs in Group 4 of MB

Gene Symbol	Fold Change	*P*-val	FDR *P*-val
LINC01419	139.78	0.0047	0.0175
OTX2-AS1	60.12	9.95E-16	2.03E-13
BLACAT1	27.67	1.13E-18	4.59E-16
DLEU2	11.58	2.25E-15	4.16E-13
LINC01355	7.09	2.23E-07	3.85E-06
MIRLET7BHG	7.01	2.03E-06	2.55E-05
PRR34-AS1	6.82	8.84E-12	6.11E-10
LINC01000	6.29	4.10E-12	3.13E-10
CKMT2-AS1	6.19	5.04E-11	2.82E-09
MIR99AHG	5.27	9.82E-07	1.38E-05

**Table 14:** Top 10 down-regulated lncRNAs in Group 4 of MB

Gene Symbol	Fold Change	*P*-val	FDR *P*-val
XIST	− 343.06	0.0287	0.0745
SOX2-OT	−31.6	1.90E-13	2.06E-11
MALAT1	−13.08	5.80E-10	2.34E-08
LINC00643	−11.64	4.39E-13	4.32E-11
LINC00844	−9.89	1.26E-05	0.0001
LRRC75A-AS1	−9.53	1.52E-08	3.87E-07
MIAT	−7.87	2.15E-10	9.90E-09
PRKAG2-AS1	−7.8	4.29E-07	6.75E-06
NR2F1-AS1	−5.98	8.89E-11	4.63E-09
PEG3-AS1	−5.74	1.92E-08	4.73E-07

Functional analysis of DE lncRNAs of Group 4 MB using IPA predicted C17orf98, ZNF426, RNF165, FBXO8, CTCF, LAYN, PYGO1, Firre, TSIX and miR-150-5pa as most significantly associated upstream regulators, while activation/inactivation of X-chromosomes, cell movement, methylation of DNA and metastasis are the most important biological functions affected in this subgroup (Tables 15 and 16). Heatmap of 5 upstream regulators is shown in supplementary Fig. 2 (Additional file 3). The two important non-canonical networks enriched with DE lncRNAs are shown in Fig. 4b and c. In network 1; AR, MYC, XIST, SP1, CCND1, and EZH2, in network 2; Histone H3, SP1, ESR1, MYC, SOX2, POU5F1, CDH1, and CEBPB are the central regulators linked with DE lncRNAs.

**Table 15:** Top 10 upstream regulators involved in DE lncRNAs in Group 4 MB

Upstream Regulator	Molecule Type	*P*-val of overlap	Target molecules in dataset
C17orf98	other	1.34E-03	XIST
ZNF426	transcription regulator	1.34E-03	XIST
RNF165	enzyme	1.34E-03	XIST
FBXO8	other	1.34E-03	XIST
LAYN	other	2.68E-03	XIST
PYGO1	other	2.68E-03	XIST
CTCF	transcription regulator	3.03E-03	TSIX,XIST
Firre	other	4.02E-03	XIST
TSIX	other	4.02E-03	XIST
miR-150-5p (and other miRNAs w/seed CUCCCAA)	mature microRNA	4.02E-03	MIAT

**Table 16:** Top 10 disease and function identified by IPA from DE lncRNAs in Group 4 MB

Categories	Diseases or Functions Annotation	*P*-val	Activation z-score
Cellular Movement	Cell movement of tumor cell lines	4.56E-06	−0.938
Gene Expression	Inactivation of mouse X chromosome	6.13E-06	
Gene Expression	Activation of mouse X chromosome	6.13E-06	
Cellular Movement	Migration of tumor cell lines	6.36E-06	−0.877
Gene Expression	Imprinting	2.27E-05	
Cell Death and Survival	Apoptosis of kidney cancer cell lines	3.30E-05	
Cancer, Organismal Injury and Abnormalities	Metastasis of tumor cell lines	1.28E-04	0.555
Cellular Movement	Invasion of tumor cell lines	1.36E-04	0.031
Cellular Development, Cellular Growth and Proliferation	Proliferation of kidney cancer cell lines	1.68E-04	
DNA Replication, Recombination, and Repair, Gene Expression	Methylation of DNA	1.82E-04	
Cell Death and Survival	Cell death of eye cell lines	3.08E-04	

### Prognostic significance of lncRNAs in different subgroups of MB

**Fig. 5:**
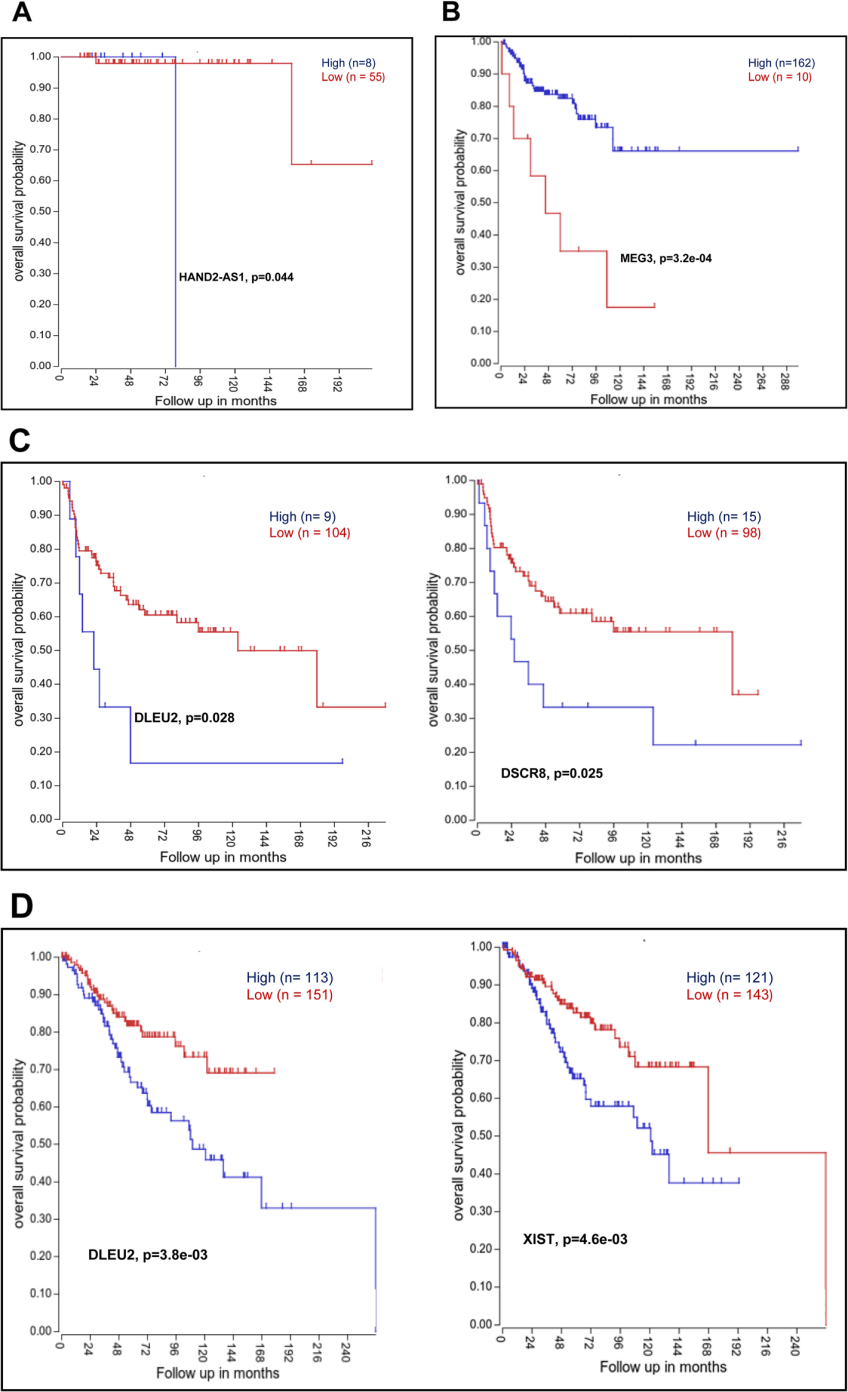
Kaplan Meier survival curves of different lncRNAs expressed in different subgroups of MB (Cavalli dataset) obtained using scan cut-off method on hgserver (https://hgserver1.amc.nl). **a**: High expression of HAND2-AS1 is associated with poor prognosis in WNT MB. **b**: Low expression of MEG3 is associated with poor prognosis in SHH MB. **c**: High expression of DLEU2 and DSCR8 are associated with poor prognosis in Group 3 MB. **d**: High expression of DLEU2 and low expression of XIST in Group 4 MB are associated with poor prognosis (*p* < 0.05)

We used a publicly available dataset GSE85217 (Cavalli dataset) to understand the prognostic significance of DE lncRNAs of different MB subgroups. As shown in Fig. 5, high expression of HAND2-AS1 is associated with poor prognosis in WNT MB. Similarly, low expression of MEG3 in SHH, high expression of DLEU2 and DSCR8 in Group 3 and high expression of DLEU2 and low expression of XIST in Group 4 are associated with poor prognosis in MB (*p* < 0.05).

## Discussion

LncRNAs are known regulators of gene expression. Disruptions in gene regulatory pathways in cancers dictate the aberrant LncRNAs expression [11–13]. Notably, almost 40% of lncRNAs are aberrantly expressed in the brain-related disorders including brain tumors. However, lncRNA expression profile in MB is largely unexplored. In this study, we have identified the lncRNA expression profile of pediatric MB subgroups and associated molecular pathways. The identified key lncRNAs require further functional validation in vitro and in vivo to explore their potential role in MB subgroup-specific manner. Here, we discuss the known cancer-relevant function of the key lncRNAs identified in MB subgroups.

EMX2OS is the most differentially expressed lncRNA in the WNT subgroup. This lncRNA is known to regulate *EMX* gene expression in the brain development [32, 33]. OTX2-AS1 (antisense strand of the *OTX2* gene) is predominantly involved in eye development [34]. High PGM5-AS1 (antisense strand of the *PGM5* gene) expression is associated with development and poor prognosis of colorectal cancer (CRC) [35]. Increased expression of DSCR8 is associated to malignant pathology and poor survival in hepatocellular carcinoma (HCC) patients [36]. LOXL1-AS1 (antisense strand of the *LOXL1* gene) is involved in the progression and metastasis of MB by regulating the PI3K-AKT signaling [27]. In addition, it is also known to play roles in the proliferation and survival of prostate cancer (PC) cells via miR-541-3p and cell cycle gene *CCND1* [37] as well as aggressive nature of glioblastoma by activating NF-kB pathway [38]. HAND2-AS1 (antisense strand of the *HAND2* gene) is overexpressed in esophageal squamous cell carcinoma (ESCC) [39] while it is downregulated in non-small cell lung cancer (NSCLC) cells [40]. TMEM51-AS1 (antisense strand of the *TMEM51* gene) is associated with renal cell carcinoma (RCC) [41]. RMST acts as a tumor suppressor in triple-negative breast cancer (TNBC) by inducing apoptosis and inhibiting proliferation/invasion and migration [42]. PART1 promotes gefitinib-resistance in ESCC by regulating the miR-129/Bcl-2 pathway [43] and also associated with PC tumorigenesis [44]. LINC00461 is involved in glioma tumorigenesis via MAPK/ERK and PI3K/AKT signaling pathways [45]. Downregulation of MEG3 is involved in the proliferation and apoptosis of PC cells by regulating miR-9-5p and its target gene *QKI-5* [46]. Downregulation of LINC00844 is associated with poor clinical outcomes and suppressed tumor progression/metastasis in PC [47]. SOX2-OT is overexpressed and promotes tumorigenesis by upregulating *SOX2* gene and activating PI3K/AKT signaling pathway in cholangiocarcinoma (CCA) [48]. SOX2-OT is also a prognostic biomarker for osteosarcoma (OS) and involved in cell survival and cancer stem cells [49]. TUNAR plays a tumor suppressive role in glioma cells by upregulating miR-200a and inhibiting Rac1 [50]. MALAT1 promotes the chemo-resistance of cervical cancer via BRWD1-PI3K/AKT pathway [51]. MALAT1 is a well-studied lncRNA in several solid and hematological cancers [52].

NEAT1 is overexpressed in most cancer types, except leukemia and myeloma, where it is down-regulated [53–55]. DLEU2 exhibits role in the proliferation and survival of laryngeal cancer cells via miR-16-1 [56]. DLEU2 is also significantly overexpressed in gastric cancer and contributes to cell proliferation [57]. TPT1-AS1 (antisense strand of the *TPT1* gene) expression is upregulated in cervical cancer and has influence on proliferation and migration [58]. HCG11 is significantly overexpressed in hepatocellular carcinoma (HCC) and genetic-silencing of HCG11 in HCC cells leads to decreased proliferation [59]. HCG11 expression is downregulated in PC and associated with poor prognosis of patients [60]. CCEPR contributes significantly in promoting cell proliferation and inhibiting apoptosis in bladder cancer [61].

BLACAT1 is overexpressed in chemo-resistant NSCLC and induces autophagy by regulating miR-17 and ATG7 pathway [62]. It also triggers proliferation/survival by regulating WNT signaling in cervical cancer [63].

XIST is elevated in bladder cancer and inhibits p53 function via binding to TET1 [64]. XIST also binds to miR-34a and elicits proliferation and tumor development in thyroid cancer [65]. XIST is an important regulator of progression and oxaliplatin-resistance in malignant melanoma [66]. MIR100HG is known to be involved in cetuximab-resistance in CRC via the β-catenin cellular pathway [67]. In addition, elevated expression of MIR100HG is correlated with poor prognosis of osteosarcoma [68]. MIAT is overexpressed in clear cell renal cell carcinoma (CCRCC) and associated with poor prognosis [69]. MIAT associates with miR-133 and contributes a role in the progression pancreatic cancer development [70]. MIAT also plays a key role in CRC tumorigenesis via miR-132/Derlin-1 axis [71]. NR2F1-AS1 (antisense strand of the *NR2F1* gene) promotes chemotherapy-resistance in HCC by regulating miR-363-ABCC1 drug-transporter pathway [72].

## Conclusions

**Fig. 6:**
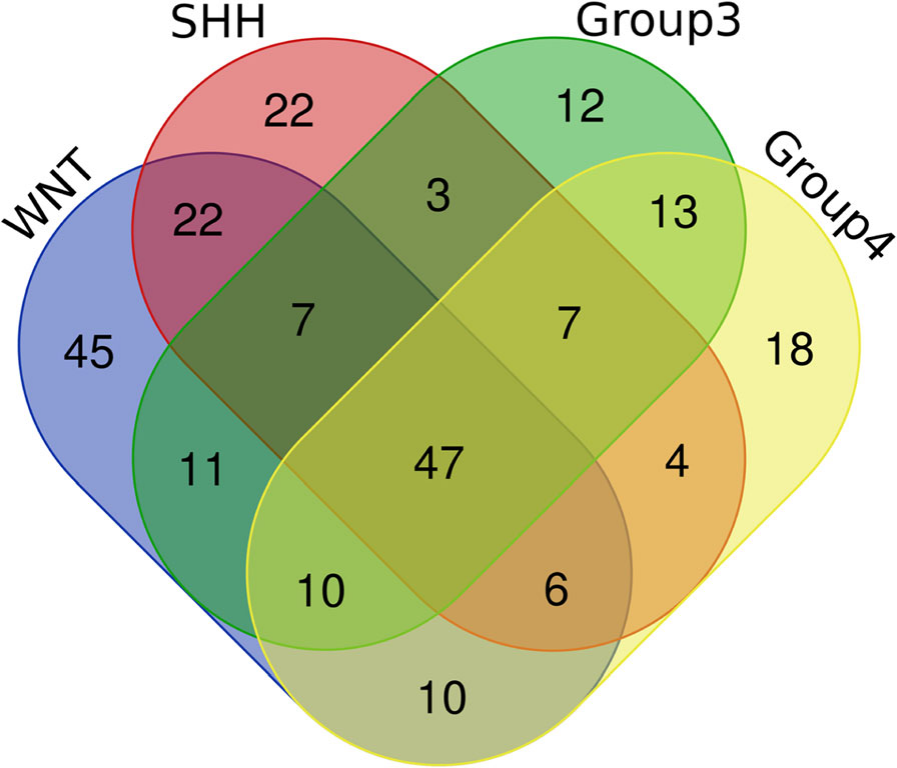
Venn diagram shows unique and overlapping lncRNAs profile in different MB subgroups. A complete list of unique lncRNAs in each subgroup can be viewed in supplementary file (highlighted in purple in Additional file 1)

We propose that the majority of DE lncRNAs in MB might have oncogenic properties as seen in other cancers (Supplementary Table S1 in Additional file 3) [73–82]. We found approximately 25% of these DE lncRNAs in MB are tumor suppressive. Also, each MB subgroup has unique and common lncRNAs in their expression profile (Fig. 6). We performed a unique lncRNAs analysis in both original datasets and validation datasets (Additional files 1 and 2). Unique lncRNAs can be validated for differential diagnosis and prognosis of MB subgroups. Common lncRNAs and associated molecules in pathways can be important therapeutic targets. We identified important lncRNAs DELU2, CASC15, LINC01355 and GAS5 are present in each subgroup and can be further explored for functional analyses in different MB subgroups. We also found SOX2, Protein kinase C delta (PRKCD), and EZH2 associated with functional networks of each subgroup and could be important drug targets. We also identified the prognostic significance of lncRNAs in different subgroups of MB.

## Supplementary information

**Additional file 1.**

**Additional file 2.**

**Additional file 3.**

## Data Availability

We used publicly available GEO datasets (https://www.ncbi.nlm.nih.gov/geo/) GSE37418, GSM1094863, GSM1094864, GSM1094865, GSM1094866, GSM1094867, GSE124814, and GSE85217 for our analyses. The gene expression data GSE85217 (Cavalli dataset) was used for survival analyses in the R2-Genomics Analysis and Visualization Platform (https://hgserver1.amc.nl/cgi-bin/r2/main.cgi).

## Abbreviations

MB: Medulloblastoma
LncRNAs: Long-non-coding RNAs
GEO: Gene Expression Omnibus
DE: Differentially expressed
mRNA: Messenger ribonucleic acid
DNA: Deoxyribonucleic acid
IPA: Ingenuity pathway analysis
ANOVA: Analysis of variance
