# Supplementary material for: Long non-coding RNA profiling of pediatric Medulloblastoma
Source: BMC Med Genomics. 2020 Jun 26;13:87. doi: 10.1186/s12920-020-00744-7 (PMC7318516; doi:10.1186/s12920-020-00744-7)
Supplement: Supplementary file 3 — Additional file 3. [file 12920_2020_744_MOESM3_ESM.docx]

**Supplementary Figure 1. Heatmap of 5 common upstream regulators of DE expressed lncRNAs in WNT and SHH subgroup.** Row and columns were clustered using correlation distance method.


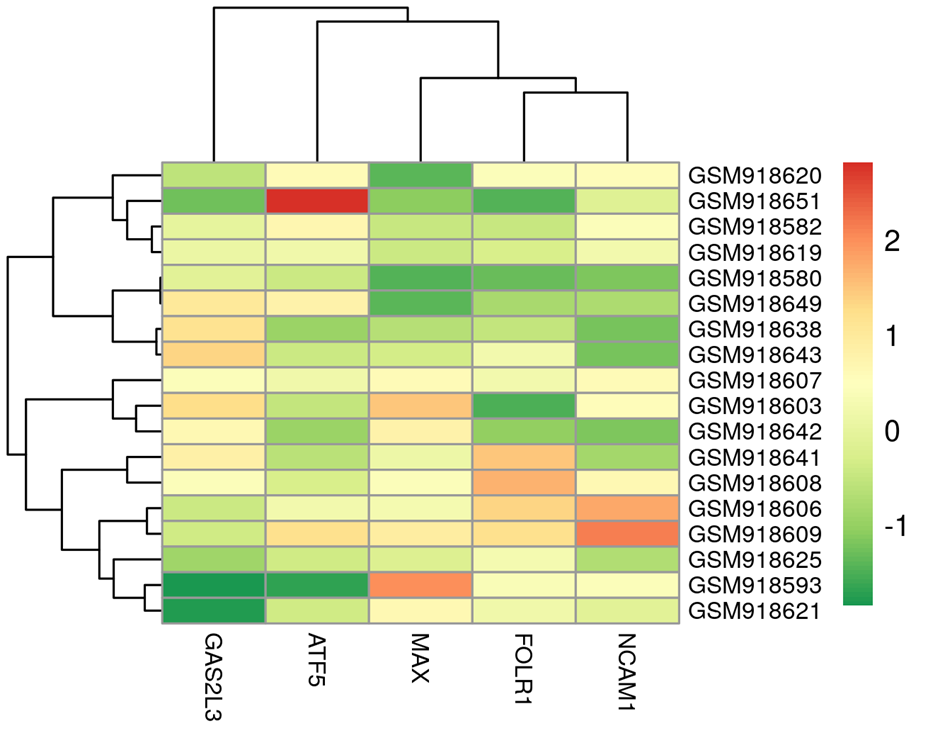


**Supplementary Figure 2. Heatmap of 5 common upstream regulators of DE expressed lncRNAs in Group 3 and Group 4 MB.** Row and columns were clustered using correlation distance method.


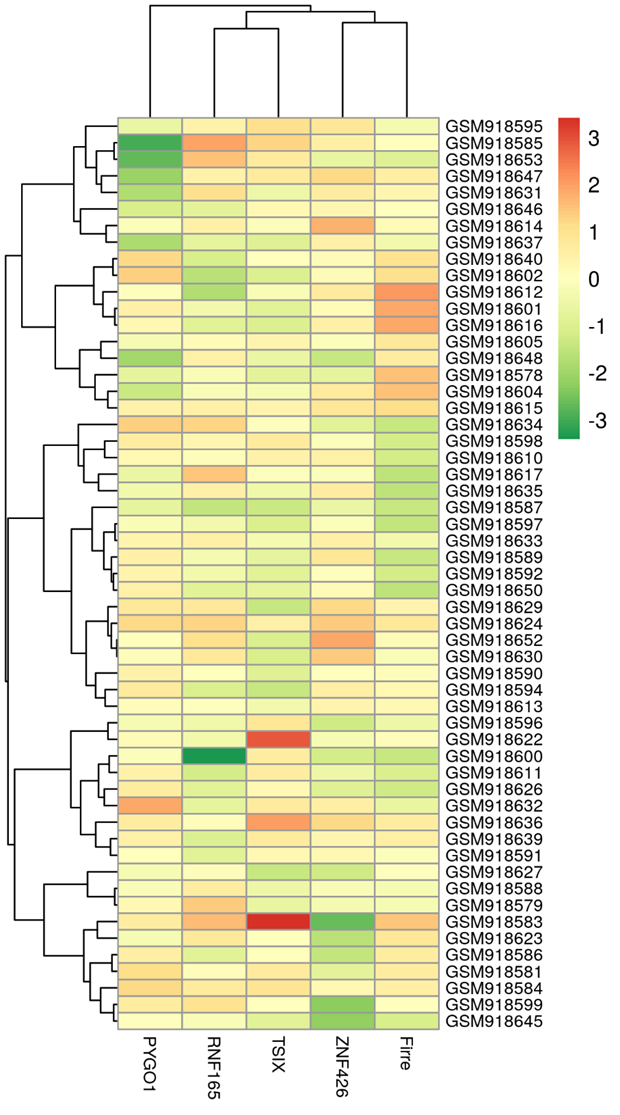


**Supplementary Table 1:** Oncogenic and tumor suppressive potential of the top identified lncRNAs across MB subgroups.

| **LncRNA** | **Functional role in cancer** | **Reference** |
| --- | --- | --- |
| EMX2OS | Oncogenic | [73] |
| OTX2-AS1 | Unknown |  |
| PGM5-AS1 | oncogenic | [35] |
| DSCR8 | Oncogenic | [36] |
| LOXL1-AS1 | Oncogenic | [27], [37] |
| HAND2-AS1 | Oncogenic | [39] |
| TMEM51-AS1 | Oncogenic | [41] |
| RMST | Tumor suppressive | [42] |
| LINC01305 | Oncogenic | [74] |
| PART1 | Oncogenic | [43], [44] |
| LINC00461 | Oncogenic | [45] |
| MEG3 | Tumor suppressive | [46] |
| LINC00844 | Tumor suppressive | [47] |
| LINC00643 | Unknown |  |
| SOX2-OT | Oncogenic | [48], [49] |
| PEG3-AS1 | Tumor suppressive | [75] |
| TUNAR | Tumor suppressive | [50] |
| MALAT1 | Oncogenic | [51], [52] |
| LINC01105 | Oncogenic | [76] |
| LINC01351 | Unknown |  |
| NEAT1 | Oncogenic | [53-55] |
| DLEU2 | Oncogenic | [56], [57] |
| PRR34-AS1 | Unknown |  |
| LINC01355 | Tumor suppressive | [77] |
| MIRLET7BHG | Unknown |  |
| CKMT2-AS1 | Unknown |  |
| SLC16A1-AS1 | Tumor suppressive | [78] |
| TPT1-AS1 | Oncogenic | [58] |
| LINC01000 | Unknown |  |
| ANP32A-IT1 | Unknown |  |
| MIR124-2HG | Unknown |  |
| HCG11 | Oncogenic | [59], [60] |
| CCEPR | Oncogenic | [61] |
| BLACAT1 | Oncogenic | [62], [63] |
| LINC00348 | Unknown |  |
| XIST | Oncogenic | [64-66] |
| MIR100HG | Oncogenic | 67, 68 |
| MIAT | Oncogenic | [69], [71] |
| TRHDE-AS1 | Oncogenic | [79] |
| LINC01419 | Oncogenic | [80] |
| MIR99AHG | Oncogenic | [81] |
| LRRC75A-AS1 | Tumor suppressive | [82] |
| PRKAG2-AS1 | Unknown |  |
| NR2F1-AS1 | Oncogenic | [72] |
